# The posterior fibulotalocalcaneal ligament complex: a forgotten ligament

**DOI:** 10.1007/s00167-020-06431-5

**Published:** 2021-01-24

**Authors:** Peter A. J. De Leeuw, Jordi Vega, Jon Karlsson, Miki Dalmau-Pastor

**Affiliations:** 1 Department of Orthopaedic Surgery, Flevoziekenhuis, Hospitaalweg 1, 1315 RA Almere, The Netherlands; 2 Department of Orthopaedic Surgery, Amsterdam UMC, Amsterdam Movement Sciences, University of Amsterdam, Amsterdam, The Netherlands; 3 Academic Center for Evidence-Based Sports Medicine (ACES), Amsterdam, The Netherlands; 4 Amsterdam UMC IOC Research Center, Amsterdam Collaboration On Health and Safety in Sports, Amsterdam, The Netherlands; 5 Human Anatomy and Embryology Unit, Department of Pathology and Experimental Therapeutics, University of Barcelona, Barcelona, Spain; 6 Foot and Ankle Unit, iMove -Tres Torres, and Hospital Quirón Barcelona, Barcelona, Spain; 7 GRECMIP (Groupe de Recherche et d’Etude en Chirurgie Mini-Invasive du Pied) Soon MIFAS (Minimally Invasive Foot and Ankle Society), Merignac, France; 8 Department of Orthopaedics, Sahlgrenska University Hospital, Sahlgrenska Academy, Gothenburg University, Gothenburg, Sweden

**Keywords:** Rouvière and canela ligament, Posterior fibulotalocalcaneal ligament, Ankle anatomy, Hindfoot endoscopy, Superior peroneal retinaculum

## Abstract

**Purpose:**

The purpose of the present anatomical study was to define the exact morphology of the posterior fibulotalocalcaneal ligament complex (PFTCLC), both for a better orientation and understanding of the anatomy, especially during hindfoot endoscopy.

**Methods:**

Twenty-three fresh frozen specimens were dissected in order to clarify the morphology of the PFTCLC.

**Results:**

In all specimens, the ligament originated from the posteromedial border of the lateral malleolus between the posterior tibiofibular ligament (superior border) and the calcaneofibular ligament (CFL), (inferior border). This origin functions as the floor for the peroneal tendon sheath. The origin of the PFTCLC can be subdivided into two parts, a superior and inferior part. The superior part forms an aponeurosis with the superior peroneal retinaculum and the lateral septum of the Achilles tendon. From this structure, two independent laminae can be identified. The inferior part of the origin has no role in the aponeurosis and ligamentous fibres run obliquely to insert in the lateral surface of the calcaneus, in the same orientation as the CFL, but slightly more posterior, which was a consistent finding in all examined specimens. The PFTCLC is maximally tensed with ankle dorsiflexion and is located within the fascia of the deep posterior compartment of the leg.

**Conclusions:**

The PFTCLC is part of the normal anatomy of the hindfoot and therefore should be routinely recognized and partly released to achieve access to the posterior ankle anatomical pathology, relevant for hindfoot endoscopy. The origin of the ligament complex forms the floor for the peroneal tendon sheath. The superior part of the origin plays a role in the formation of an aponeurosis with the superior peroneal retinaculum and the lateral septum of the Achilles tendon.

## Introduction

Defining and understanding human anatomy is important for adequate, safe and reproducible surgery without iatrogenic damage to our patients [4, 6, 7]. A specific anatomical hindfoot structure has become apparent since the introduction of the hindfoot endoscopy by van Dijk et al. [17]. It was a continuous finding that a strong and broad fascial like structure had to be penetrated in order to be able to reach the level of the posterolateral subtalar joint to subsequently identify the flexor hallucis longus (FHL) and the ankle joint [18]. It seemed to attach to the posterolateral talar process and had the appearance of a ligament. In the modern anatomy books, this endoscopic finding could not be identified [11, 20].

**Fig. 1:**
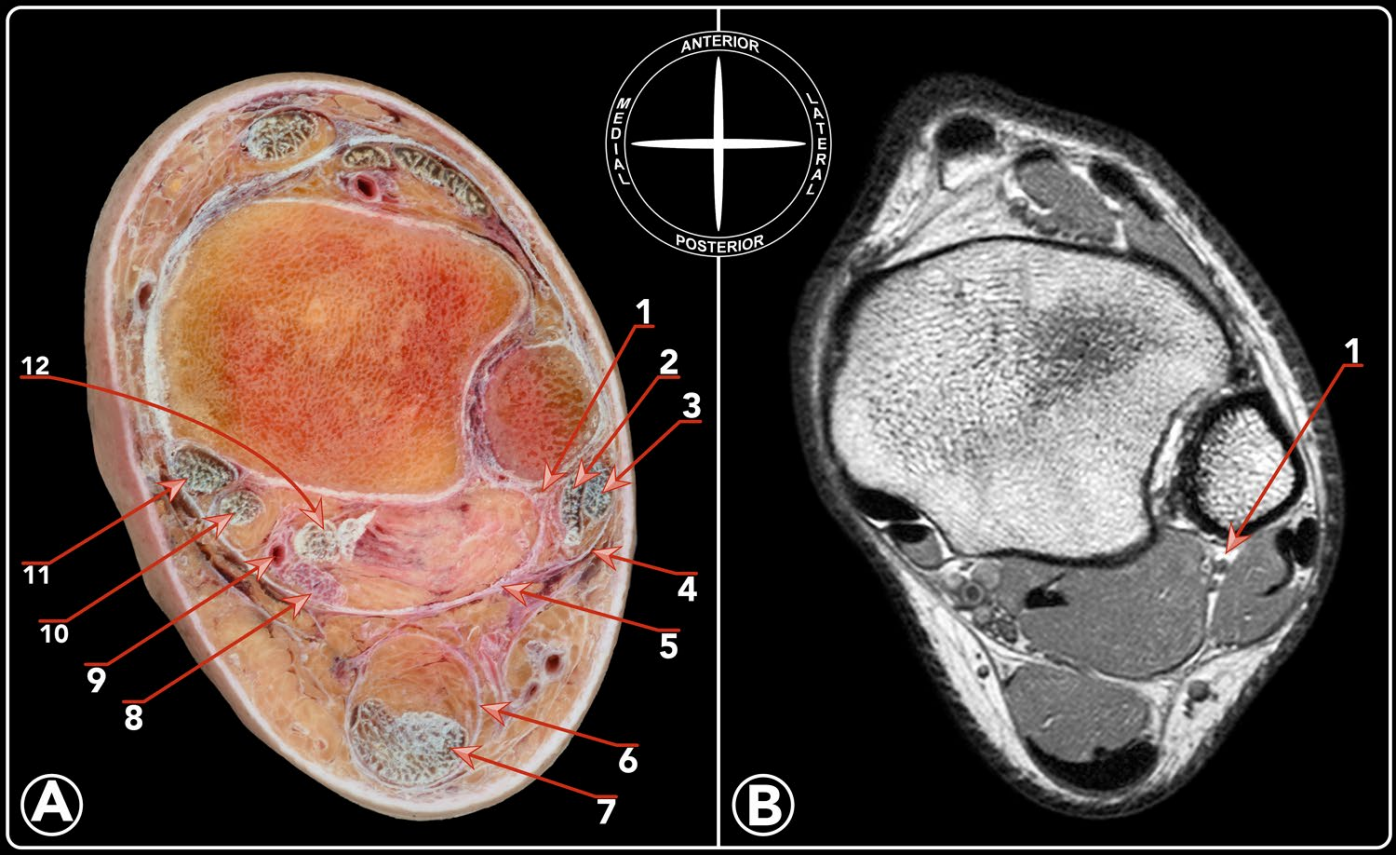
**a** Cross section at the level of the tibial pilon. **b** Comparative MRI to highlight the location of the PFTCLC. 1. PFTCLC. 2. Peroneus brevis tendon. 3. Peroneus longus tendon. 4. Superior peroneal retinaculum. 5. Superior lamina of the PFTCLC. 6. Fascia of the superficial posterior compartment. 7. Achilles tendon. 8. Tibial nerve. 9. Posterior tibial artery. 10. Flexor digitorum longus tendon. 11. Tibialis posterior tendon. 12. Flexor hallucis longus tendon

Dujarier described the posterior fibulocalcaneal ligament in 1924 as an anatomical anomaly [5]. In 1932, Rouvière and Canela Lazaro described the posterior fibulotalocalcaneal ligament (PFTCL), being a specialized part of the fascia of the deep posterior compartment of the leg [12]. They described this structure as an extrinsic ligament that occupies the posterior and posterolateral corner of the ankle joint [12] (Fig. 1). Sarrafian described some anatomical aspects of this ligament, resembling the work by Rouvière and Canela Lazaro [13].

Rouvière and Canela Lazaro concluded that the PFTCL was a portion of the deep fascia of the leg, which forms part of the tunnel of the flexor hallucis longus tendon and the peroneal tendons sheath. The frequency of this finding as a well-defined ligament was 60%, in 20% only a thin and weak ligament was identified and in the remaining 20% of the cases the ligament was reported absent [12].

Several studies presumed that the PFTCL was possibly partially responsible for subtalar and ankle stability; however, no anatomical studies were conducted to provide evidence in this matter [1, 8, 9, 14]. For safe and reproducible surgery detailed knowledge of all the relevant anatomy is essential. Therefore, the purpose of the present anatomical study was to define the exact morphology of the ligament, both for a better orientation and understanding of the anatomy, especially during hindfoot endoscopy. Moreover, its possible anatomical function in the hindfoot was assessed based on the anatomical orientation. Given the constant endoscopic occurrence of this ligament, it was hypothesized that this ligament is part of normal hindfoot anatomy.

## Methods

Twenty-three fresh frozen ankles (eleven males and twelve females) from below-the-knee specimens of the Anatomy Department of the University of Barcelona, Spain, were dissected in detail to examine the morphology and possible variations of the PFTCLC. The specimens were from Caucasian origin with a mean age of 83 years (range 56–96 years). Specimens were excluded in case of deformities or scars due to previous surgery.

A digital caliper (Australian measuring instruments 0–200 mm) was used in order to measure the width of the origin and insertion of the PFTCLC, performed by the dissecting anatomist. The dissections were digitally recorded (Nikon D810, 105 mm Micro Nikon F 2.8 lens, raw format) to allow for comparison of the morphological ligamentous characteristics in the specimens.

**Fig. 2:**
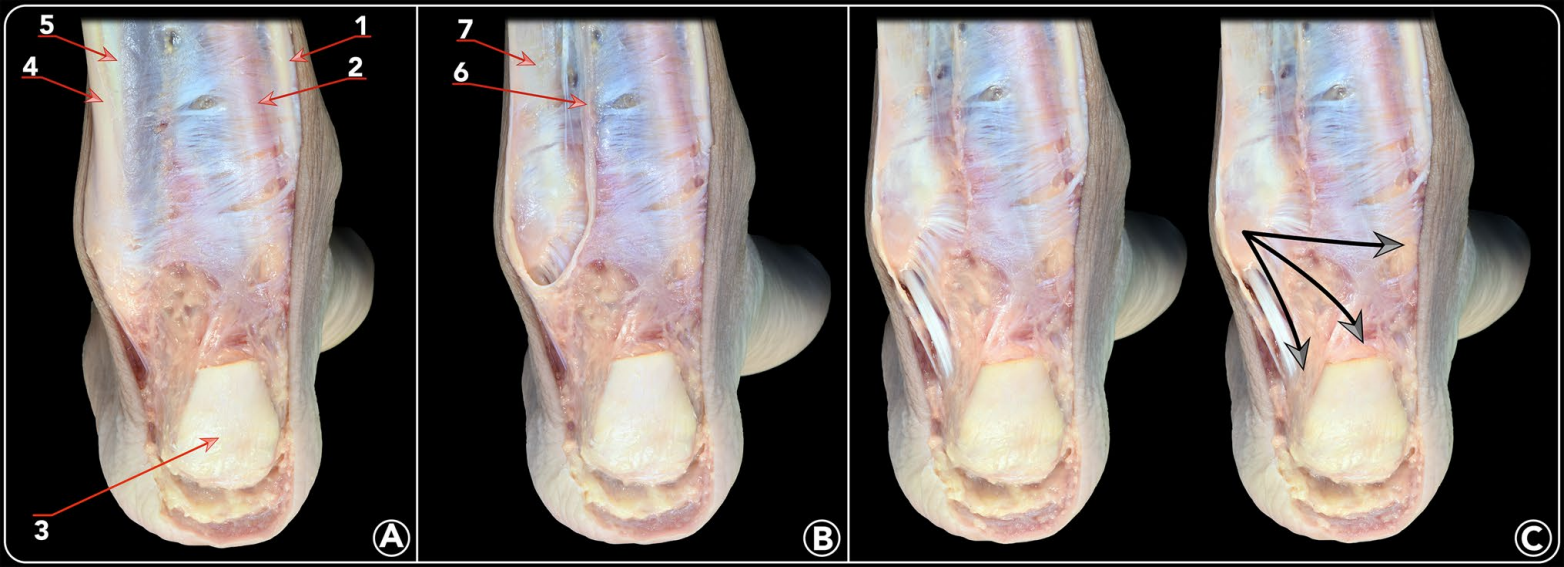
Anatomical dissection showing the methodology used in this study. **a** Posterior view of a left ankle with the triceps surae resected and the Achilles tendon partially resected. 1. Flexor digitorum longus tendon. 2. Tibial nerve. 3. Achilles tendon. 4. Peroneus longus tendon. 5. Peroneus brevis tendon. **b** The lateral compartment of the leg has been exposed and the peroneal tendons have been resected. 6. Medial border of the peroneal tendon sheath/intermuscular septum. 7. Posterior peroneal diaphysis. **c** Dissection has been advanced until clear visualization of the three laminae (superior, middle and inferior) of the PFTCLC is possible

The technique performed was a plane-per-plane anatomical dissection by an experienced anatomist (either PG or M D-P). The methodology used is highlighted in Fig. 2; the skin and subcutaneous fat tissue were removed from the posterior aspect of the ankle, exposing the Achilles tendon and, medially and laterally, the fascia of the deep posterior compartment. Relations of the deep fascia and the lateral septum of the Achilles tendon were noted. The Achilles tendon and Kager’s fat pad were subsequently removed, while the medial septum of the Achilles tendon was left intact. Subsequently, the sheath of the peroneal tendons was opened from proximal to distal and the superior peroneal retinaculum was released leaving the lateral peroneal tendon septum unattended. Then, the peroneal tendons were removed in order to be able to carefully assess the origin of the PFTCLC. The calcaneofibular ligament (CFL) and the superficial component of the posterior tibiofibular ligament were identified and their relation to the PFTCLC was assessed. The course of the PFTCLC in all directions and planes was studied in detail and each insertion site was marked.

The insertions of the PFTCLC were subsequently released from medial to lateral to assess its relation to the posterior neurovascular bundle (tibial nerve, posterior tibial artery and accompanying veins), flexor hallucis longus tendon, posterolateral talar process, posterior talofibular ligament and intermalleolar ligament.

## Results

At anatomic dissection, the PFTCLC was identified in all the twenty-three fresh frozen specimens. In all specimens, the ligament originated from the posteromedial border of the lateral malleolus. The origin had an average width of 2.5 (range 1.8–3.2) cm, proximally limited by the superficial component of the posterior tibiofibular ligament (superior border of the PFTCLC), and distally by the CFL (inferior border of the PFTCLC). In three specimens, the superior border was partly continuous with the superficial component of the posterior tibiofibular ligament. In all ankles, the inferior border of the PFTCLC origin was clearly separated from the origin of the CFL.

**Fig. 3:**
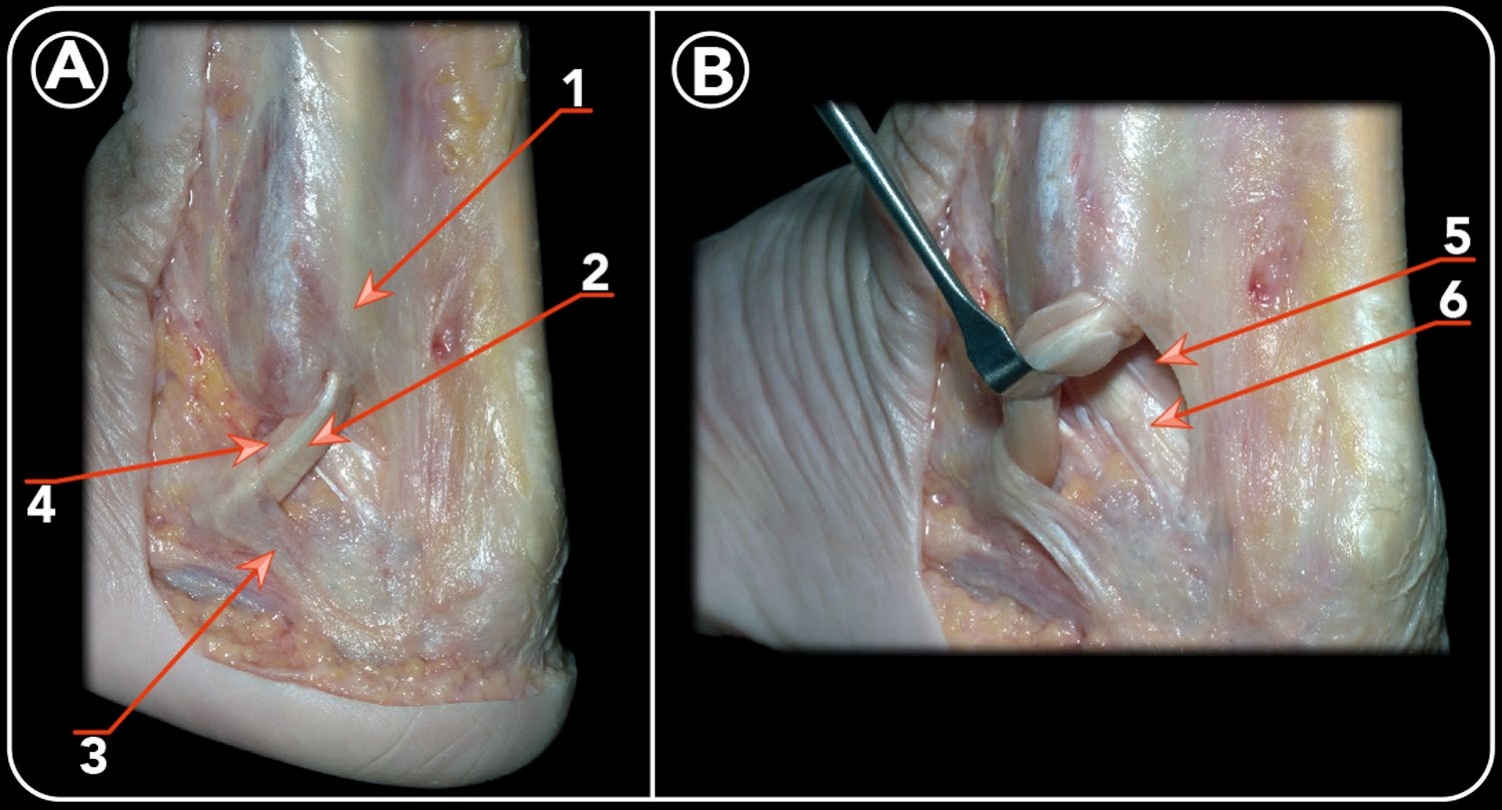
Posterolateral view of a left ankle demonstrating the relation between the PFTCLC and the peroneal tendons. **a** 1. Superior peroneal retinaculum. 2. Peroneus longus tendon. 3. Inferior peroneal retinaculum. 4. Peroneus brevis tendon. **b** The peroneal tendons are displaced anteriorly in order to show the floor of its tunnel, which is the PFTCLC. 5. Inferior band of the PFTCLC. 6. CFL

The peroneus longus and brevis tendons are located in the lateral compartment of the leg and share a common peroneal tendon sheath. At the level of the distal fibula, the synovial sheath runs through a fibro-osseous tunnel. The anterolateral border of the tunnel was formed by the retromalleolar groove. The posterior border of the tunnel is formed by the superior peroneal retinaculum (Fig. 3).

**Fig. 4:**
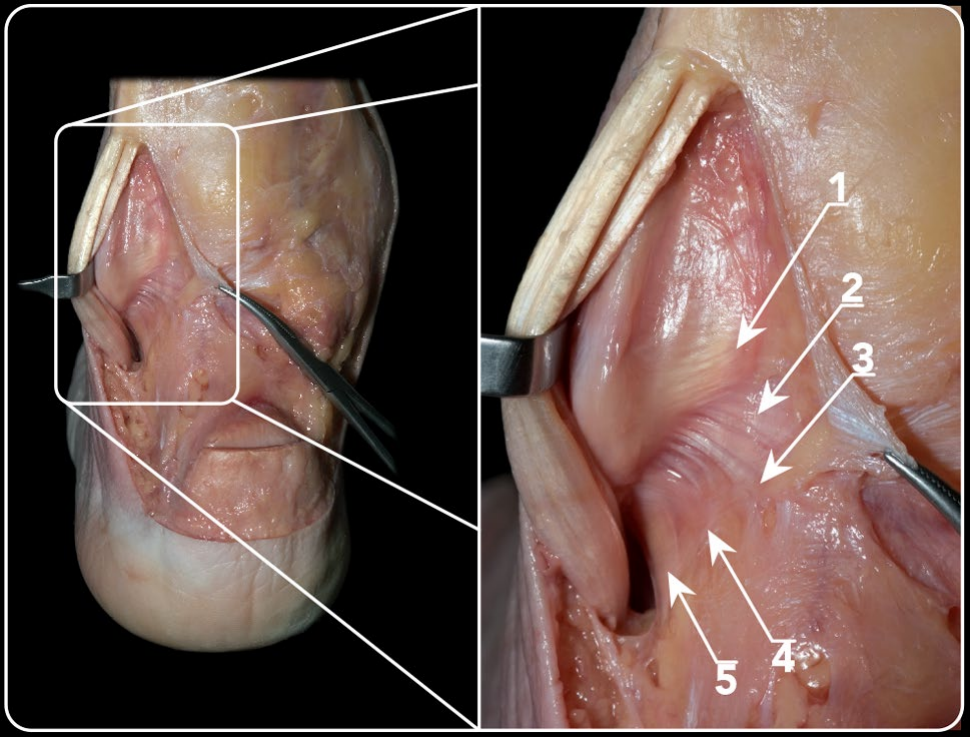
Posterior view of a dissection highlighting the limits of the PFTCLC. 1. Posterior tibiofibular ligament (superior limit of the PFTCLC). 2. Superior lamina of the PFTCLC. 3. Middle lamina of the PFTCL. 4. Inferior lamina of the PFTCLC. 5. CFL (inferior limit of the PFTCLC)

The origin of the PFTCLC can be subdivided into a superior and inferior part. The superior part blends together with the lateral septum of the Achilles tendon and the superior peroneal retinaculum, forming a so-called aponeurosis. In all dissected specimens, this aponeurosis is a rigid structure. The aponeurosis forms the medial border of the peroneal tendon sheath. Dependent of the level at the distal fibula, from proximal to distal, the anteromedial border is formed by the superficial component of the posterior tibiofibular ligament, the PFTCLC and the CFL, respectively (Fig. 4). This was a constant finding in every specimen.

From the inferior part of the PFTCLC origin, just proximal to the CFL, an independent lamina, not playing a role in the aponeurosis, is present inserting directly in the lateral surface of the calcaneus. This lamina inserts in an oblique direction, posteriorly to the origin of the CFL. The insertion is, however, in close approximation to the insertion of the aponeurosis in the lateral surface of the calcaneus. This independent lamina was observed in all the dissected specimens and is named the inferior lamina.

The lateral septum of the Achilles tendon, part of the aponeurosis, runs in a vertical downward direction inserting in the superolateral surface of the calcaneus. Since the aponeurosis is located proximal to this calcaneal insertion, it is not just the septum inserting but rather is a combined insertion of the lateral Achilles tendon septum, the superior peroneal retinaculum and the superior part of the PFTCLC origin. From the aponeurosis, in addition to the vertical lamina inserting in the lateral border of the calcaneus, two other independent laminae are identified; one ran in a medial direction (superior lamina or band), the other in an oblique direction (middle lamina or band).

**Fig. 5:**
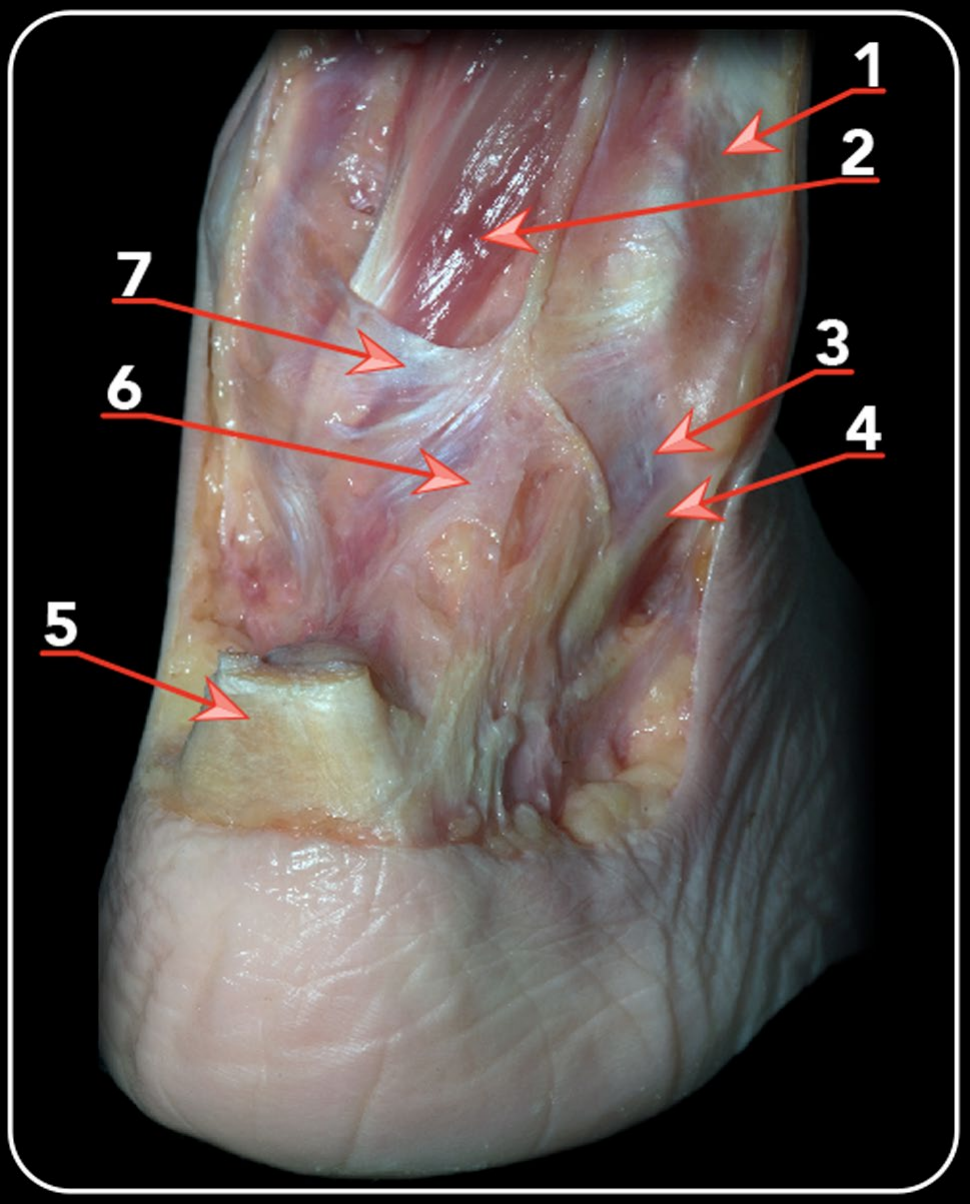
Posterolateral view of a dissected right ankle demonstrating the relation between the PFTCLC and the flexor hallucis longus. 1. Posterior peroneal diaphysis. 2. Flexor hallucis longus. 3. Inferior lamina of the PFTCLC. 4. CFL 5. Achilles tendon. 6. Middle lamina of the PFTCLC. 7. Superior lamina of the PFTCLC

The superior lamina, which inserted in the posterolateral border of the medial malleolus, posteriorly and inferiorly to the insertion of the intermalleolar ligament, was identified. From the aponeurosis, this lamina runs medially to cover the flexor hallucis longus tendon (Fig. 5). Medially to the flexor hallucis longus, the lamina splits, one running anteriorly and the other posteriorly to the posterior neurovascular bundle. These subdivisions of the superior lamina were clearly visualized in nineteen specimens.

**Fig. 6:**
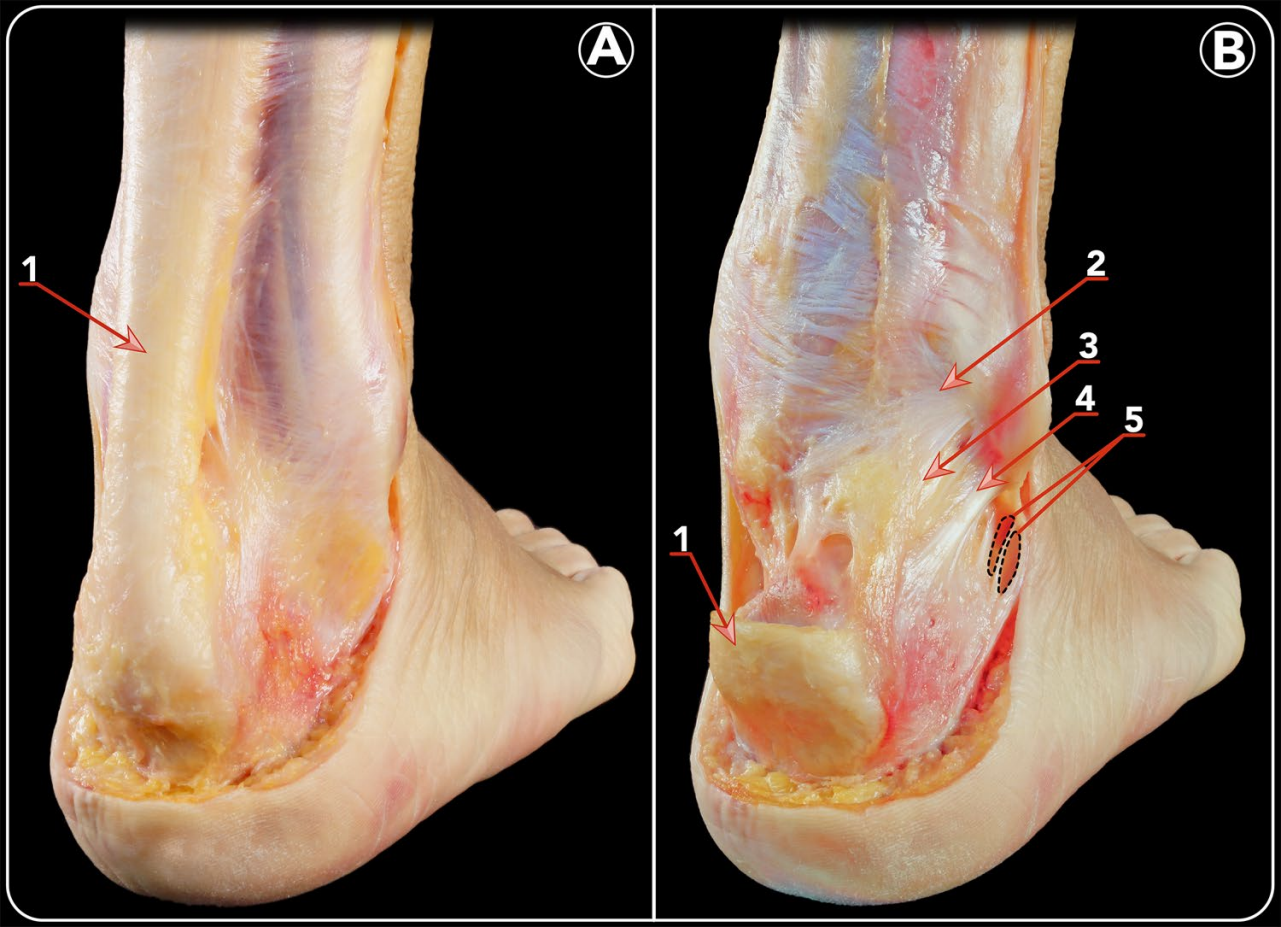
Posterolateral view following the dissection of a left ankle of a dissection with the Achilles tendon in place (**a**) and then with the Achilles and peroneal tendons cut (**b**). 1. Achilles tendon. 2. Superior lamina of the PFTCLC. 3. Middle lamina of the PFTCLC. 4. Inferior lamina of the PFTCL. 5. Tunnels for peroneus brevis and longus tendons

A second obliquely orientated lamina, originating from the aponeurosis and inserting in the posterolateral talar process, posteriorly to the origin of the posterior talofibular ligament. This lamina was is called the middle lamina. In all the cases, the posterior talofibular ligament was present. The middle lamina is located posteriorly, as compared to the superior lamina and was present in all the dissected specimens (Fig. 6).

The CFL, the origin of the PFTCLC, the posterior talofibular ligament, the aponeurosis and each of the 3 mentioned laminae (superior, middle and inferior) were tight with the ankle in dorsiflexion. Ankle dorsiflexion slightly increased (some degrees), while each of the laminae was subsequently released, although this was not objectively tested in this study. Its effect on the varus and valgus stability on the subtalar joint could not specifically be assessed in this research protocol.

**Fig. 7:**
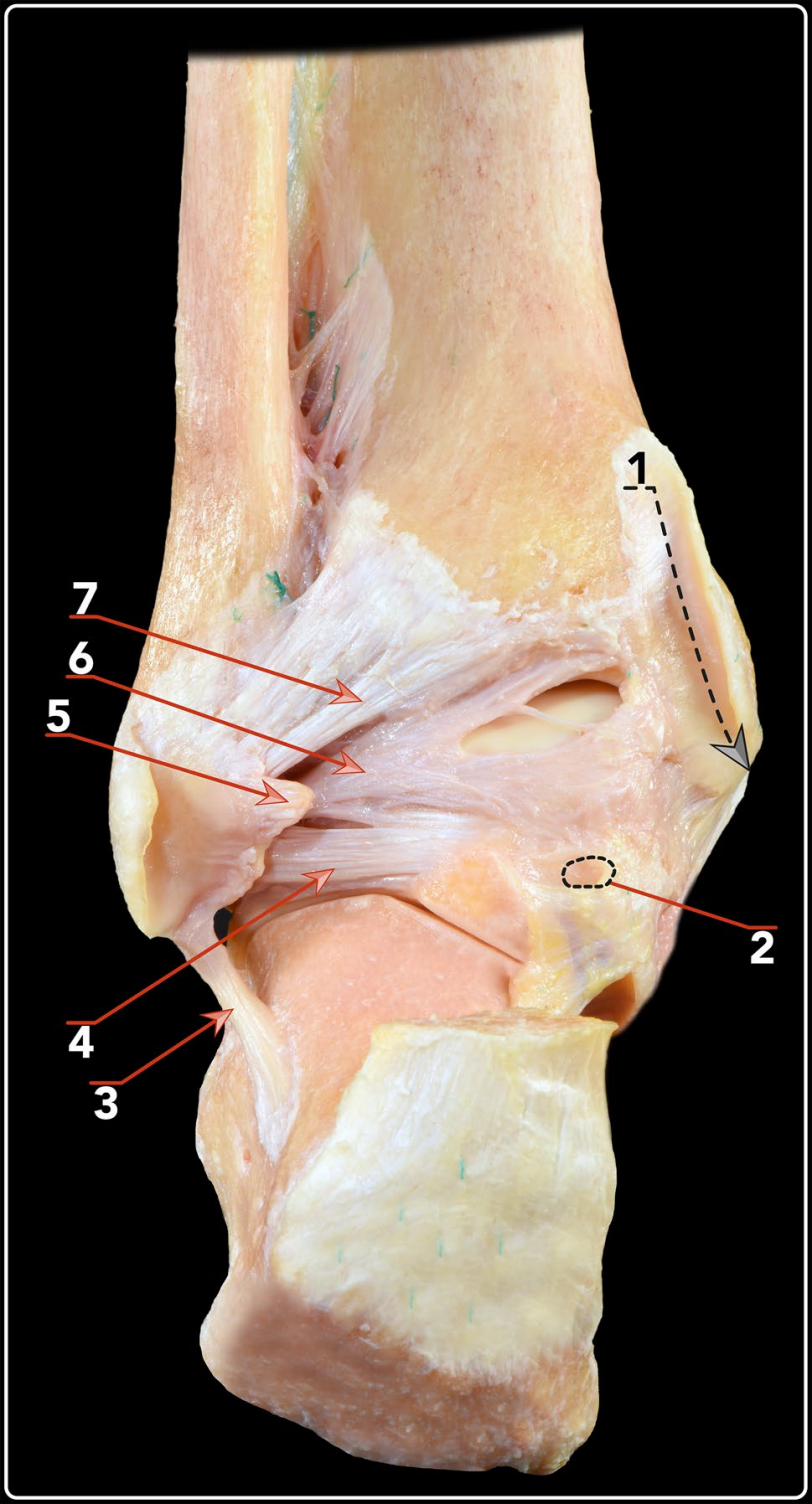
Posterior view of an osteo-articular dissection of the ankle, where the fibular origin of the PFTCL can be observed. 1. Pathway of tibialis posterior tendon. 2. Tunnel for flexor hallucis longus tendon. 3. Calcaneofibular ligament. 4 Posterior talofibular ligament. 5. Fibular origin of PFTCL. 6. Intermalleolar ligament. 7. Posterior tibiofibular ligament

The posterior talofibular ligament and the intermalleolar ligaments are posteriorly covered by the origin of the PFTCLC and medially and inferiorly by each of the laminae originating from the aponeurosis (Fig. 7). Between these layers, a fat tissue pad is observed. The posterior talofibular ligament originated from the medial surface of the lateral malleolus (fibular fossa), coursing horizontally and it inserted in the posterolateral talar process, anteriorly to the insertion of the superomedial lamina of the PFTCLC. The main fibres from the intermalleolar ligament ran from the insertion in the medial border of the lateral malleolus, superiorly and anteriorly to the origin of the PFTCLC, inserting in the lateral–inferior border of the medial malleolus. The insertion was in close correlation with the insertion of the superior lamina of the PFTCLC. The posterior talofibular ligament and the intermalleolar ligament were identified in all specimens and their relation to the PFTCL was found to be consistent.

## Discussion

**Fig. 8:**
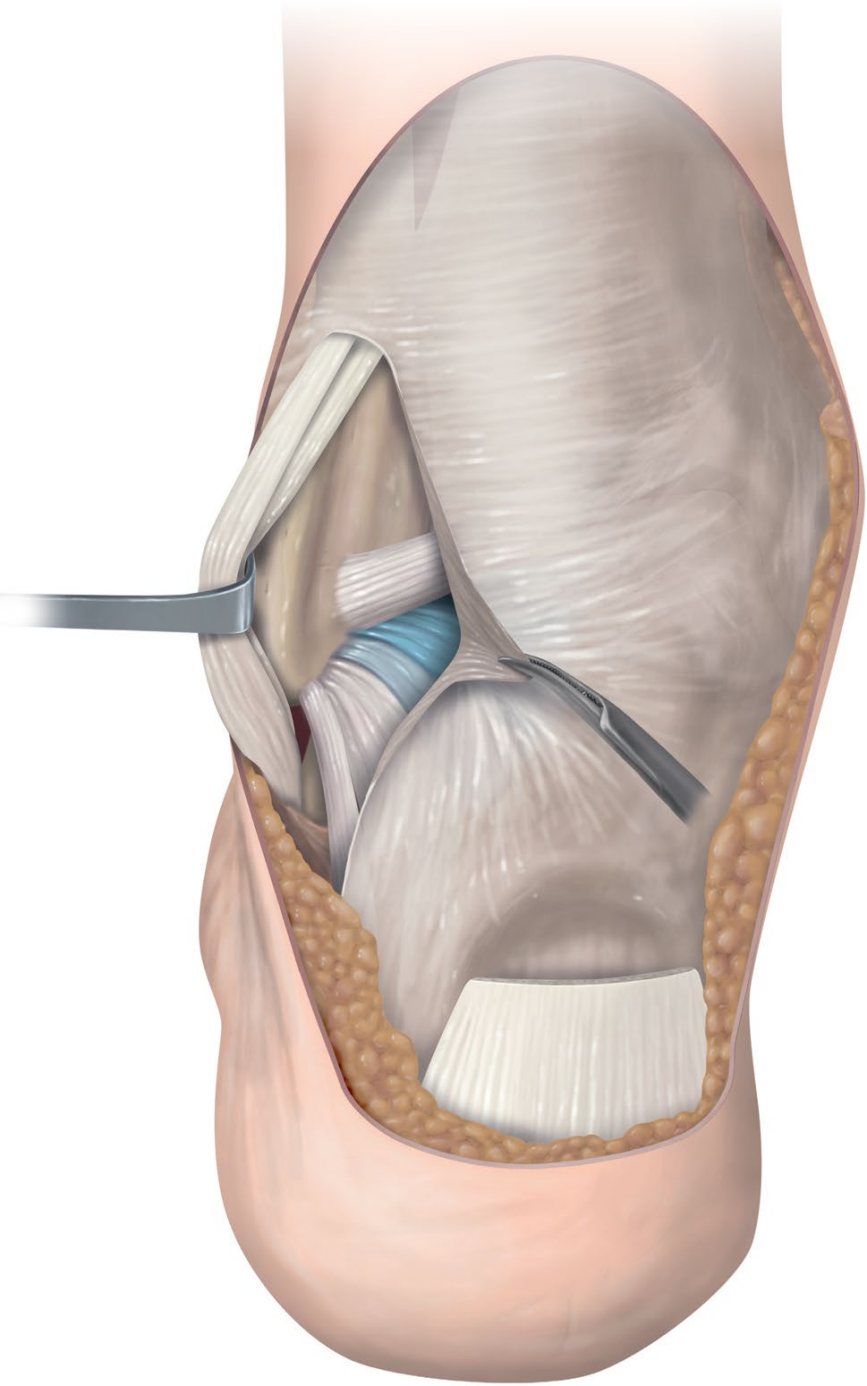
Drawing illustrating the anatomy of the PFTCLC, with the peroneal tendons retracted. The posterior tibiofibular ligament is observed as the superior limit of the PFTCLC, while the CFL is its inferior limit. The three laminae of the PFTCLC are highlighted in colour

The most important finding of the present study is that the PFTCLC is an extrinsic ligament, located in the posterolateral area of the ankle (Fig. 8). In fact, this ligament is a specialized part of the fascia of the deep posterior compartment, which is a constant finding in all examined specimens. Clinically, the PFTCLC can be regarded as part of the normal hindfoot anatomy. Due to its insertional characteristics, the PFTCLC limits ankle dorsiflexion. It is located posterior to the tendons of the muscles in the deep posterior compartment of the leg.

The origin of the PFTCLC was found to be located at the posteromedial border of the lateral malleolus. The average width of the origin was 2.5 (range 1.8–3.2) cm. Proximally, the origin of the PFTCLC is limited by the posterior tibiofibular ligament and distally by the CFL. Rouvière and Canela Lazaro also concluded that this ligament had its insertion at the medial border of the lateral malleolus, but found variations mainly at the inferior border of the origin [12], which was not reproduced in this study.

At the level of the distal fibula, the peroneal tendon sheath runs through a fibro-osseous tunnel. The anterolateral border of the tunnel was formed by the fibular sulcus and the medial border was formed by the aponeurosis (lateral septum of the Achilles tendon, superior peroneal retinaculum and the superior part of the PFTCLC origin). The posterior border of the tunnel was formed by the superior peroneal retinaculum. Dependent of the level at the distal fibula, from proximal to distal, the anteromedial border was formed by the superficial component of the posterior tibiofibular ligament, the PFTCLC and the CFL, respectively. This observation has not been previously specified, although Rouvière and Canela Lazaro described its anatomical orientation anteriorly to the peroneal tendons [12].

One lamina (inferior) was constantly recognized as a structure running from the distal part of the origin and inserting in the lateral surface of the calcaneus. The insertion is oblique, posteriorly and separated from the origin of the CFL. This lamina is identical to the posterior fibulocalcaneal ligament, as described by Dujarier in 1924 [5]. In most of their dissections, Rouvière and Canela Lazaro found this lamina inserting transversely in almost the entire superior surface of the calcaneus, although in some of their examined specimens the insertion was also at the lateral surface of the calcaneus as an oblique insertion, covering both the superior and lateral calcaneal surface [12].

The lateral septum of the Achilles tendon and the superior peroneal retinaculum inserted in the lateral surface of the calcaneus. Their close correlation, especially in combination with the PFTCLC could however not be found in the literature. The peroneal tendons wrap around the lateral malleolus from posterior to anterior. At this level, the lateral septum of the Achilles tendon, the superior peroneal retinaculum and the superior part of the PFTCLC origin blended together to form an aponeurosis. The aponeurosis inserted in the lateral surface of the calcaneus, lateral and posterior to the origin of the CFL. The insertion in the lateral surface of the calcaneus is identical to the description of Davis et al., however, these researchers consider the superior peroneal retinaculum being an independent structure [3]. Sarrafian also showed a figure in his book with a short comment about the joining of the PFTCLC with the superior peroneal retinaculum; nevertheless, the exact morphological characteristics were not provided [13]. Rouvière and Canela Lazaro concluded that the PFTCLC was in close connection with the fibrous peroneal tendon sheath, but soon separated from it [12]. This separation was not recognized in any of our specimens.

From the aponeurosis, two constant laminae originated; a superior and middle lamina. The middle lamina was observed in all the dissected specimens inserting in the posterolateral talar process. It was continuous with the origin of the talocalcaneal ligament and was orientated posteriorly to the origin of the posterior talofibular ligament. Given its anatomical location, this lamina needs to be at least partially released to allow access to the posterior ankle and subtalar joint during the hindfoot endoscopy. In addition, a study about the role of the PFTCLC in dorsiflexion restriction has recently been published [10].

In all the specimens, the PFTCLC was clearly recognized covering the posterior talofibular ligament and the intermalleolar ligament, separated by a fat tissue pad, and the ankle joint capsule. Performing hindfoot endoscopy requires passage of the PFTCLC, the fat tissue pad and joint capsules to visualize the posterior anatomical structures. The flexor hallucis longus tendon should routinely be visualized prior to pathology assessment, since the posterior neurovascular bundle is located directly medial to it [16, 19]. This tendon separates the different laminae of the PFTCLC. The superior lamina is part of the flexor hallucis longus retinaculum. The middle lamina, inserting in the posterior talar process, prevents the visualisation of the flexor hallucis longus tendon and should therefore be released and/or partly removed during hindfoot endoscopy.

Stephens et al. concluded that the PFTCLC possessed a particularly strong stabilizing effect on the ankle and for a lesser extent on subtalar joint stability. The effect on the subtalar stability increased with the ankle in dorsiflexion [15]. In all specimens, the aponeurosis as well as the laminae were tensed in dorsiflexion, as was also described by Rouvière and Canela Lazaro [12]. Our finding of the existence of an aponeurosis is supported by the fact that the superior peroneal retinaculum was described being tensed in dorsiflexion [15]. Since Stephens et al. probably used the morphological characteristics of the ligament as provided by Rouvière and Canela Lazaro, the exact values concerning the individual role of the PFTCLC in stability characteristics on the subtalar and ankle joint are therefore questionable.

Interesting however is the independent lamina directing from the inferior part of the PFTCLC not taking part in the aponeurosis. Presumably, this lamina has an independent functional role in stabilizing the subtalar joint. Since the PFTCLC only needs to be released partially during hindfoot endoscopy, it may be assumed that the PFTCLC will not have influence on the ankle and/or subtalar stability, especially since the aponeurosis will remain intact. Evidence in this direction can however not be provided, which is a limitation of this study.

The CFL, the posterior talofibular ligament, the aponeurosis and all laminae of the PFTCLC were tight with the foot in dorsiflexion in all assessed specimens. The ankle dorsiflexion slightly increased, while each of the PFTCLC laminae was subsequently released. Therefore, it is assumed that one of the functions of the PFTCLC is to limit the ankle dorsiflexion. Some patients following hindfoot endoscopy develop ankle stiffness which might potentially be addressed to the PFTCLC. Stiffness could result from either scarring and slight retraction of the PFTCL following the surgical operative technique or the formation of fibrotic adhesions between the posterior structures of the ankle and the PFTCLC. Further research might need to focus on this subject.

One of the limitations of the study is the lack of biomechanical testing of the ligament, which could further have added to the functional understanding of the PFTCLC. Another important fact to consider is the complex anatomy of the PFTCLC and the surroundings structures. For this reason, dissection of the specimens by experienced anatomists is recommended [2].

In conclusion, the PFTCLC is a constant part of normal hindfoot anatomy, being a specialized part of the deep crural fascia. The PFTCLC origin forms the floor for the peroneal tendon sheath at the level of the distal fibula. The superior part of the ligament’s origin forms an aponeurosis with the superior peroneal retinaculum and the lateral septum of the Achilles tendon, altogether inserting in the lateral surface of the calcaneus playing a part in the limitation of ankle dorsiflexion. The PFTCLC should routinely be recognized and subsequently be partly released to assess the hindfoot during hindfoot endoscopy. Following the recognition, it should be partly released to allow access to the posterior ankle and subtalar joint during the hindfoot endoscopy.

The authors would like to suggest including this ligament to the posterior anatomical ankle slides in anatomy books.
